# Mitochondrial derived peptide MOTS‐c prevents the development of heart failure under pressure overload conditions in mice

**DOI:** 10.1111/jcmm.17551

**Published:** 2022-09-25

**Authors:** Peng Zhong, Jianye Peng, Yewen Hu, Jun Zhang, Caijie Shen

**Affiliations:** ^1^ Department of Cardiology Renmin Hospital of Wuhan University Wuhan China; ^2^ The Second Affiliated Hospital, Department of Cardiovascu lar Medicine, Hengyang Medical School University of South China Hengyang China; ^3^ Department of Cardiology Ningbo First Hospital Ningbo China; ^4^ Department of Emergency, Tongji Medical Collage, Wuhan Central Hospital Huazhong University of Science and Technology Wuhan China

**Keywords:** heart failure, inflammation, MOTS‐c, oxidative stress, pressure overload

## Abstract

MOTS‐c, a mitochondrial‐derived peptide (MDP), has been shown to have multiple biological activities such as antioxidation, anti‐inflammation, and anti‐apoptosis properties. In the present study, we aimed at evaluating the therapeutic effect of MOTS‐c peptide in an animal model of heart failure. The heart failure mouse model was made by transverse aortic constriction (TAC) operations. The MOTS‐c peptide was administrated subcutaneously by using an osmotic pump. At the end of the animal experiment, cardiac function was evaluated by echocardiography, and heart tissues were subjected to histological and molecular analysis. In vitro cultured H9C2 cells were used to test the effects of MOTS‐c overexpression on cell death in response to H_2_O_2_ stimulation. Our study showed that MOTS‐c peptide attenuated TAC‐induced cardiac dysfunction and remodelling. In addition, the MOTS‐c peptide reduced the inflammatory response and upregulated the antioxidant capacity, coupled with the activation of the AMPK pathway in the heart of the TAC mouse model. In in vitro cultured cardiac cells, overexpression of MOTS‐c was shown to activate the AMPK pathway and protect cell apoptosis in response to H_2_O_2_ stimulation. Taken together, our study suggested that MOTS‐c peptides may have therapeutic potential in treating HF.

## 1 INTRODUCTION

Heart failure (HF) is a syndrome resulting from the inability of the cardiac pump to meet the energy requirements of the body. Multiple mechanisms have been implicated in the pathogenesis of HF, including inflammation, increased oxidative stress, energy metabolism abnormalities, cardiac cell apoptosis, interstitial fibrosis, and mitochondrial dysfunction.^1^ Although there is a significant improvement in morbidity and mortality over the last decades, the current treatment of HF remains suboptimal. Therefore, new therapeutic strategies are required.

Mitochondrial‐derived peptides (MDPs) are small bioactive peptides encoded by short open‐reading frames in mitochondrial DNA. To date, eight MDPs have been identified, all of which are regulators of metabolism.^2^ Of the known native MDPs, MOTS‐c has most consistently been reported to have metabolic‐protective properties in multiple animal models of metabolic stress.^3^ Recent studies have also shown that MOTS‐c could exert cytoprotective effects through anti‐oxidative stress, anti‐inflammatory, and anti‐apoptosis mechanisms.^4^, ^5^ For instance, under stress conditions, MOTS‐c could translocate to the nucleus and bind directly to the promoter regions of Nrf2‐response genes, such as HO‐1 and NQO‐1, to promote antioxidant gene expression.^6^ Peritoneal injection of MOTS‐c has been shown to inhibited systemic and tissue inflammatory response in a lipopolysaccharide (LPS)‐induced acute lung injury mouse model.^7^ Collectively, these results suggested that MOTS‐c may be a potential therapeutic agent in treating HF.

In the present study, we investigated the therapeutic potential of MOTS‐c in treating HF induced by pressure overload in mice. Our results showed that the administration of MOTS‐c peptide could protect against the adverse remodelling and dysfunction of the heart under pressure overload conditions. In addition, overexpression of MOTS‐c in in vitro cardiac cells could protect cell apoptosis in response to oxidative stress. Taken together, our study suggested that the MOTS‐c peptide may have therapeutic potential in treating HF.

## 2 METHODS

### 2.1 Ethics statement

The present study was conducted according to the “Guide for the Care and Use of Laboratory Animals” (NIH Publication No.85–23, revised in 2010). The animal experiments were approved by the Laboratory Animal Welfare and Ethics Committee of Renmin Hospital of Wuhan University. Male C57BL/6 mice (8–10 weeks old) were purchased from the Institute of Laboratory Animal Science, Chinese Academy of Medical Science. The animals were housed and raised in a pathogen‐free environment with an appropriate temperature and humidity.

### 2.2 Transverse aortic constriction operations and animal treatment

Transverse aortic constriction (TAC) operations were performed to establish the HF model according to the previously published literature.^8^ During the operations, mice were anaesthetised with 2% isoflurane in oxygen and ventilated at a tidal volume of 0.3 ml at 80 breaths/min. The aorta was tied against a 28‐gauge needle with a 7–0 silk suture. Four weeks post surgery, the mice were subjected to echocardiography followed by division into two groups: one group was administrated with the human MOTS‐c peptide (obtained from Genscript Corp, 5 mg/kg/day) subcutaneously for 4 weeks by using the ALZET osmotic minipumps (mode 1004, ALZET); The other group was just administrated with saline as a control group. At the end of the experiments, all mice were euthanized with the administration of an overdose of sodium pentobarbital (200 mg/kg; i.p.), following which the cardiac tissues were collected and snap‐frozen in liquid nitrogen.

### 2.3 Echocardiography

Cardiac function was evaluated by performing echocardiography under the anaesthetised condition with 1.5% isoflurane after TAC surgery at various time points indicated in the manuscript. This analysis was performed using the MyLab 30CV system (Biosound EsaoteInc.) equipped with a 15‐MHz probe. M‐mode tracings derived from the short axis of the left ventricle at the level of papillary muscles were recorded. Parameters were recorded for five consecutive cardiac cycles and averaged for final data analysis. LV morphology was primarily assessed by measuring the left ventricular internal diastolic diameter (LVIDd) and left ventricular ejection fraction (LVEF).

### 2.4 Histological analysis

Mouse hearts were fixed in 10% formalin, followed by embedding in paraffin according to standard histological protocols. The hearts were then transversely sliced into 5‐μm‐thick sections in the region close to the apex. Sections of heart samples were stained with Sirius red to evaluate the extent of interstitial and perivascular fibrosis. Interstitial fibrosis was quantified as the percentage of Sirius red‐stained area in 10 random fields. Perivascular fibrosis was quantified by the ratio of the Sirius red‐stained area surrounding the vessel divided by the total vascular area. Apoptosis was assessed using the TdT‐mediated dUTP nick end labeling (TUNEL) assay kit (ab66108 from Abcam), and slides were viewed using a fluorescence microscope (Olympus). Percentage of TUNEL‐positive (apoptotic) cells was calculated by dividing the number of TUNEL‐positive cells by the total number of cells in four separate fields. Images were quantified using the Image‐Pro Plus 6.0 (Media Cybernetics Corporation).

### 2.5 Real‐time quantitative PCR

Total RNA was isolated from various organs using TRIzol reagent (Invitrogen), reverse‐transcribed to cDNA, and analysed by quantitative PCR using Lightcycler 480 SYBR Green 1 Master Mix (Roche) and the Applied Biosystems VII7 (Life Technologies) for collagen I, collagen II, and CTGF (connective tissue growth factor), TNF‐⍺ (Tumour necrosis factor‐⍺), IL‐6 and ICAM‐1 (intercellular cell adhesion molecule‐1) and Nrf2 (Nuclear factor erythroid 2‐related factor 2), HO‐1 (Heme oxygenase 1), and NQO‐1 (NAD[P]H Quinone Dehydrogenase 1) using the gene‐specific primers. The primer sequences are indicated in Table 1. All reactions were normalized using β‐actin as the endogenous control. Data were analysed using the 2^−∆∆Ct^ method.^9^ The fold changes of each target mRNA expression relative β‐action under experimental or control conditions were calculated based on the threshold cycle (CT) as r = 2^−Δ(ΔCT)^, where ΔCT = CT (target) − CT (β‐action) and Δ(ΔCT) = ΔCT (experimental) − ΔCT (control).

**TABLE 1:** PCR primer sequence

Gene	Species	Forward primer	Reverse primer
TNF‐α	Mouse	AAGCCTGTAGCCCACGTCGTA	GGCACCACTAGTTGGTTGTCTTTG
IL‐6	Mouse	GAGGATACCACTCCCAACAGACC	AAGTGCATCATCGTTGTTCATACA
ICAM‐1	Mouse	GTGATGCTCAGGTATCCATCCA	CACAGTTCTCAAAGCACAGCG
β‐Actin	Mouse	GGCTGTATTCCCCTCCATCG	CCAGTTGGTAACAATGCCATGT
Collagen1	Mouse	CCTCAGGGTATTGCTGGACAAC	CAGAAGGACCTTGTTTGCCAGG
Collagen II	Mouse	GGGTCACAGAGGTTACCCAG	ACCAGGGGAACCACTCTCAC
CTGF	Mouse	GGCCTCTTCTGCGATTTCG	GCAGCTTGACCCTTCTCGG
HO‐1	Mouse	GATAGAGCGCAACAAGCAGAA	CAGTGAGGCCCATACCAGAAG
NQO‐1	Mouse	AGGATGGGAGGTACTCGAATC	TGCTAGAGATGACTCGGAAGG

### 2.6 Western blot analysis

Total proteins were extracted from ventricles and H9C2 cells. The protein concentration was determined using the BCA protein assay kit. Fifty micrograms of protein were used for SDS‐PAGE electrophoresis. And, the proteins were transferred to a polyvinylidene fluoride membrane, which were incubated with various primary antibodies overnight at 4°C. pAMPK antibody (#2535), AMPK antibody (#2532) and Tubulin antibody (#2144) were obtained from Cell signalling Technology; HO‐1 antibody (ab13248) was from Abcam. After incubation with a secondary HRP‐conjugated antibody, signals were visualized with ECL (Bio‐Rad) reagent. The amounts of the proteins were analysed using Image J software and normalized to their respective control.

### 2.7 Cell culture and treatments

H9C2 cells embryonic rat heart‐derived cell lines were obtained from the Shanghai Institute of Biochemistry and Cell Biology and cultured in DMEM/F12 medium (Gibico) supplemented with 5% FBS, 100 U/ml of penicillin, and 100 mg/ml of streptomycin in a humidified atmosphere of 5% CO_2_ at 37°C.

### 2.8 Cell transfection and viability assay

H9C2 cells were transfected with an empty vector or MOTS‐c expression vector by using lipofectamine‐3000 (Invitrogen) following the manufacturer's transfection protocol. Following the transfection, cells were cultured in low FBS concentration (2%) for another 3 days. Then, H_2_O_2_ at different concentrations was added in culture medium for 24 h, followed by cell viability analysis by using the Cell counting kit‐8 (CCK‐8; Dojindo).^1^ The absorbance of each well was measured at 450 nm wavelength using a microplate reader (Bio Tek Instruments Inc.). The relative survival rate was quantified by using the absorbance value in each well divided by the absorbance value of the well transfected with control vector without H_2_O_2_ stimulation. The absorbance value of the well transfected with control vector without H_2_O_2_ stimulation was set as 1.

### 2.9 Statistical analysis

Data are represented as the mean ± SD. Data were analysed by anova, followed by Tukey's Multiple Comparison Test. All statistical analyses were performed in GraphPad pro5.0 software (GraphPad Software Inc.). The difference with *p* < 0.05 was considered statistically significant.

## 3 RESULTS

### 3.1 Treatment with MOTS‐c peptide attenuated cardiac dysfunction under pressure overload conditions

**FIGURE 1:**
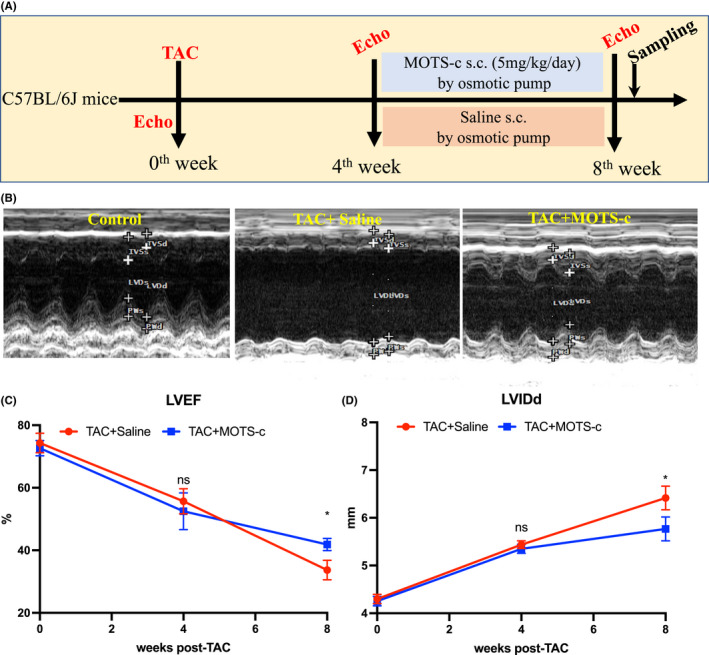
Administration of MOTS‐c peptide prevented cardiac dysfunction in TAC‐induced HF mice. (A) Schematic image of the experimental design. (B) Representative echocardiograms showing short‐axis M‐mode 2D echocardiography performed at the end of the animal experiment. (C) LVEF at different time points post‐TAC. (D) LVIDd at different time points post‐TAC. LVEF, left ventricular ejection fraction; LVIDd, left ventricular internal diameter during diastole; TAC, transverse aortic constriction. Data are represented as the mean ± SEM, *n* = 6 in each group. *p < 0.05; **p < 0.01

To determine the role of MOTS‐c on HF, we evaluated the therapeutic effects of MOTS‐c peptide on the development of HF induced by transverse aortic constriction (TAC) surgery in mice. The schematic image of the animal experiment is shown in Figure 1A. The MOTS‐c peptide was administrated by using an osmotic pump and was started 4 weeks post‐TAC surgery. The dose of the MOTS‐c peptide used is based on the previous published works, in which such as a dose was shown to exert its biological effects in animal mouse model.^10^, ^11^ Cardiac function was evaluated by echocardiography at different time points during the experiment as indicated in Figure 1A. Throughout TAC surgery, we found that cardiac dysfunction and dilation were progressively induced 4 weeks post TAC surgery, suggesting the successful establishment of the cardiac pressure overload mouse model (Figure 1C,D). Without drug intervention, cardiac dysfunction was more apparent 8 weeks post TAC, while treatment with MOTS‐c significantly attenuated the progress of cardiac function and structure deterioration as evidenced by the change of LVEF and LVIDd (Figure 1B,D). It should be noted there was no apparent difference in the heart rate and body temperature between different groups during echocardiography analysis. (Data not shown). Collectively, these results indicate a therapeutic potential of MOTS‐c on pressure overload‐induced HF.

### 3.2 Treatment with MOTS‐c peptide attenuated cardiac fibrosis and apoptosis in pressure overload‐induced HF mouse model

**FIGURE 2:**
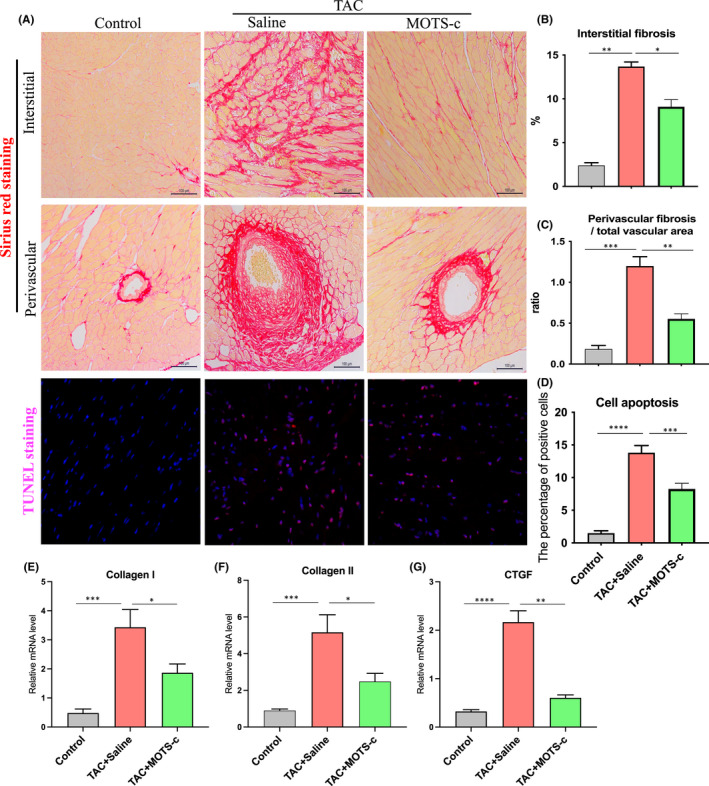
Administration of MOTS‐c peptide attenuated cardiac fibrosis and apoptosis in transverse aortic constriction (TAC)‐induced HF mice. (A) Representative images of Sirius red staining and TUNEL staining of the cardiac tissues. The quantitative data for the interstitial and perivascular fibrosis are shown in B and C; The quantitative data for the TUNEL positive cells in the heart are showed in D. (E–G) The mRNA level of collagen I, collagen II, and CTGF in the heart tissues. Data are represented as the mean ± SEM, *n* = 5–6 in each group, *p < 0.05; **p < 0.01; ***p < 0.001; ****p < 0.001

Chronic hemodynamic pressure overload could result in accumulation of fibrosis in the interstitial and perivascular space in the left ventricle (LV).^12^ We then assessed the degree of cardiac fibrosis by using Sirius red, which could bind to collagen fibrils. As shown from Figure 2A–C, remarkable fibrosis was observed in the interstitial and perivascular space of LV in response to TAC compared to the control group without TAC surgery. However, TAC‐induced fibrosis was significantly attenuated by MOTS‐c treatment (Figure 2A–C). Consistently, analysis of the mRNA expression of fibrosis‐related genes in the LV also showed a similar result in the levels of collagen I, collagen II, and CTGF in the heart tissues (Figure 2E–G). Collectively, these results suggest that MOTS‐c peptide could protect the development of cardiac fibrosis under pressure overload conditions. Next, we evaluated the extent of cell apoptosis in the heart by using the TUNEL assay. As shown from Figure 2A,D, a significant amount of apoptosis cells was observed in the TAC + Saline group compared to the control group as evidenced by the positive cells with TUNEL staining in the heart (Figure 2A,D). However, the fraction of apoptotic cells was significantly reduced by MOTS‐c treatment (Figure 2A,D). Collectively, these results indicate that MOTS‐c peptide could protect the heart from fibrosis and apoptosis under pressure overload conditions.

### 3.3 Treatment with MOTS‐c peptide attenuated cardiac inflammatory response and upregulated cardiac antioxidant capacity in the heart under pressure overload conditions

**FIGURE 3:**
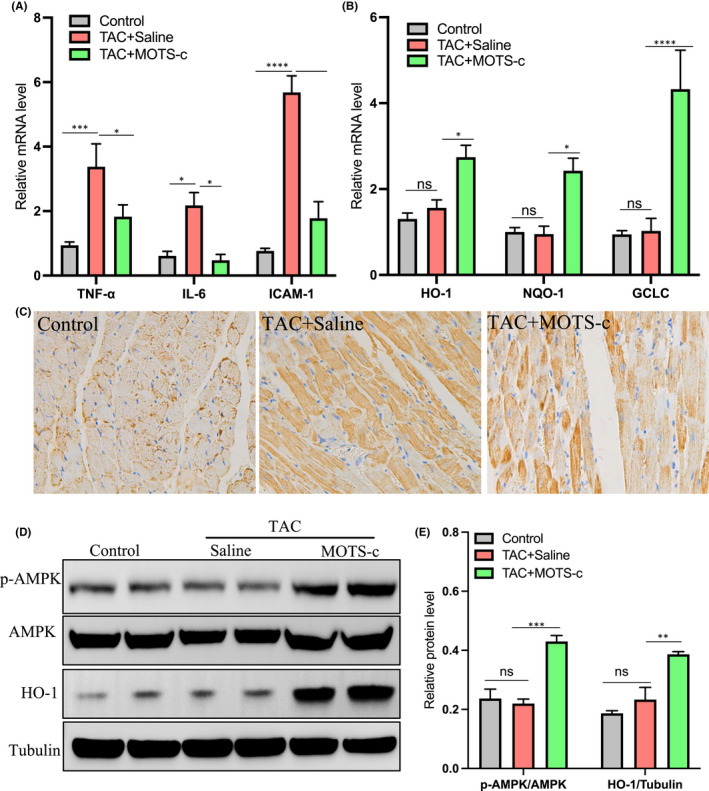
Administration of MOTS‐c peptide attenuated cardiac inflammation and upregulated cardiac antioxidant capacity in pressure overload‐induced HF mice. (A) RT‐qPCR analysis of the mRNA level of inflammatory cytokines such as TNF‐⍺, IL‐6, and ICAM‐1 in the heart tissue. (B) RT‐qPCR analysis of the mRNA level of antioxidant genes such as HO‐1, and NQO‐1 and GCLC in the heart tissue. (C) Representative images of TNF‐α immunohistochemical staining in the heart tissue. (D) Western blot analysis of pAMPK, AMPK, and HO‐1 in the heart tissue, the tubulin was used a loading control. (E) Statistical data for the pAMPK/AMPK and HO‐1/Tubulin were determined based on D. *n* = 5–6 in each group, *p < 0.05; ***p < 0.01; ****p < 0.001

Inflammation is involved in the initiation and progression of heart failure.^13^ The MOTS‐c peptide has been shown to have anti‐inflammatory capacity. We then evaluated the mRNA levels of inflammatory genes in the heart tissues. Interestingly, the expression of the inflammatory genes such as TNF‐α, IL‐1β, and IL‐6 were significantly upregulated in the TAC + Saline group compared to the control group, suggesting the presence of inflammatory response in the heart under pressure overload conditions (Figure 3A). However, all these changes were significantly reduced in the presence of MOTS‐c (Figure 3A). Immunohistochemical staining of TNF‐α further confirmed the presence of inflammatory cytokines in the TAC‐treated heart, and treatment with MOTS‐c peptide significantly reduced the intensity of TNF‐α staining (Figure 3C). Taken together, these results suggested that MOTS‐c could inhibit the inflammatory response in the heart under pressure overload conditions.

Nrf2 is a transcription factor that coordinates the basal and stress‐inducible activation of a vast array of antioxidant genes and represents a crucial regulator of the cellular defence mechanisms against oxidative stress.^14^ As MOTS‐c has been reported to have the capacity to promote antioxidant gene expression of Nrf2‐related genes, we then evaluated Nrf2‐regulated downstream antioxidant genes in the heart tissues. Interestingly, the antioxidant genes such as HO‐1, NQO‐1, and GCLC showed no apparent change after TAC surgery but were significantly upregulated after drug intervention with MOTS‐c (Figure 3B). In addition, the protein level of HO‐1 in the heart was also found to be upregulated after drug intervention with MOTS‐c peptide (Figure 3D). As activation of AMPK (AMP‐activated protein kinase) is a key point in mediating the antioxidant effects of MOTS‐c both in vitro and in vivo,^15^ we then evaluated the activity of AMPK in the heart. As shown in Figure 3D, the protein level of AMPK in its phosphorylated state (p‐AMPK/AMPK) was also significantly elevated in the MOTS‐c treated hearts but not in TAC‐treated or control group, suggesting that treatment of MOTS‐c induced activation of the AMPK pathway in the heart. Collectively, these results suggested that MOTS‐c could upregulate the antioxidant capacity of the heart under pressure overload conditions.

### 3.4 Overexpression of human MOTS‐c protected cell apoptosis in H9C2 cells in response to H_2_O_2_ stimulation

**FIGURE 4:**
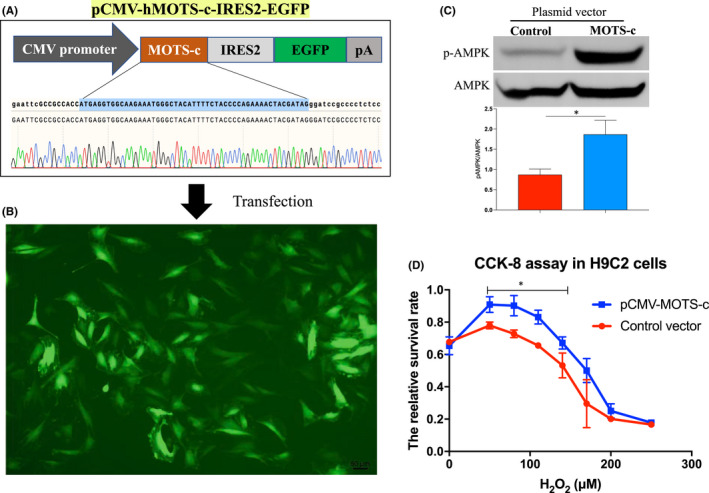
MOTS‐c peptide protected cell apoptosis in H9C2 cells in response to oxidative injury. (A) Schematic images of human MOTS‐c (hMOTS‐c) expressing plasmid vector (pCMV‐hMOST‐c‐IRES2‐EGFP), the sequencing data of MOTS‐c coding sequence was indicated. (B) Representative fluorescent images of H9C2 cells after transfected with hMOTS‐c expressing plasmid vector for 3 days. (C) Western blot analysis of the protein level of phosphorylated AMPK (pAMPK) and AMKP in the cells. (D) Cell survival analysis by using CCK8 assay in H9C2 cells in response to H_2_O_2_ stimulation after transfected with MOTS‐c expression vector or blank control vector. *p < 0.05

To investigate the cellular protective effects of MOTS‐c in cardiac cells against oxidative injury in vitro, we evaluated the effects of MOTS‐c overexpression on H_2_O_2_‐induced cell death in vitro cultured cardiac H9C2 cells. As shown in Figure 4A, we first constructed a MOTS‐c‐expressing plasmid, which also allow simultaneous expression of EGFP separately. The coding sequence of human MOTS‐c was highlighted in the sequencing data as shown in Figure 4A. Then, we transfected the H9C2 cells with the MOTS‐c expression vector or empty vector (control vector) for 3 days, followed by the transfection efficiency evaluation via fluorescent microscopy. As shown in Figure 4B, bright green fluorescence can be observed in cells transfected with plasmid vectors, suggesting that MOTS‐c could be expressed in H9C2 cells. In addition, we also evaluated the effects of MOTS‐c overexpression on the activation of the AMPK pathway in H9C2 cells. Interestingly, the protein levels of activated AMPK in its phosphorylated form at the Thr172 sites were significantly elevated after MOTS‐c vector transfection for 3 days compared to that in the control group, suggesting that overexpression of MOTS‐c in H9C2 cells could induce the activation of the AMPK pathway (Figure 4C). Then, these transfected cells were further subjected to different doses of H_2_O_2_ stimulation for 24 h, followed by cell survival analysis by using CCK‐8 assay. Interestingly, we found that cells transfected with MOTS‐c expression vector were more resistant to cell death in response to H_2_O_2_ treatment than the control group (Figure 4D). These results suggested that overexpression of MOTS‐c could protect cells against oxidative injury in cardiac cells.

## 4 DISCUSSION

Despite existing therapies for HF, the current 1‐year mortality rate remains high.^16^ As such, new strategies to treat this disease are needed. MOTS‐c is a 16‐amino‐acid peptide encoded in the mitochondrial genome. Recent studies have shown that MOTS‐c has multiple biological activities, such as metabolism regulation, anti‐inflammation, and antioxidant capacity. In the present study, we evaluated the effects of subcutaneous administration of human MOTS‐c peptides on the development of HF in mice under chronic pressure overload conditions. Very excitingly, we found that treatment of MOTS‐c significantly delayed the development of cardiac dysfunction and structural dilation under pressure overload conditions. In addition, reduced inflammatory markers and upregulated antioxidant genes were also observed in MOTS‐c‐treated heart tissues. Therefore, our research demonstrated a therapeutic potential of MOTS‐c peptide in preventing the development of HF in mice under chronic pressure overload conditions.

Recent studies suggested an anti‐inflammatory and anti‐oxidant effect of MOTS‐c. For example, microarray analysis showed that in vitro treatment of HEK293T cells with MOTS‐c peptide for 72 h could significantly reduce the expression of inflammation‐associated genes and cytokines.^15^ Peritoneal injection of MOTS‐c was reported to reduce the basal levels of circulating IL‐6 and TNF‐α in mice fed with a normal diet and inhibit systemic and tissue inflammatory response in lipopolysaccharide (LPS)‐induced acute lung injury mouse model.^7^, ^15^, ^17^ In addition, intraperitoneal administration of MOTS‐c was also shown to reduce pro‐inflammatory cytokines and elevate the level of anti‐inflammatory cytokines in the mouse formalin test model.^18^ Other studies showed that MOTS‐c could increase cellular resistance against oxidative stress. For instance, in response to glucose/serum restriction, oxidative stress, or pro‐oxidant, all of which can increase intracellular reactive oxygen species (ROS) levels, and MOTS‐c could rapidly translocate to the nucleus and bind to nuclear DNA to promote antioxidant genes expression.^6^ In addition, stably overexpressing MOTS‐c in HEK293 cells could protect against glucose/serum restriction‐induced cell death compared to cells transfected with empty vector control.^6^ Collectively, these results suggested an antioxidant and anti‐inflammatory property of the MOTS‐c peptide. In the present study, we further showed that treatment with MOTS‐c could remarkably reduce the inflammatory cytokines and upregulate antioxidant genes in the heart of the HF mouse model. As inflammation and oxidative stress play key roles in the development of HF, so the reduced inflammation and upregulated antioxidant capacity mediated by MOTS‐c possibly contributed to the improved cardiac function under pressure overload conditions.

AMP‐activated protein kinase (AMPK), an energy sensor, has pleiotropic cardioprotective effects and plays a critical role in the progression of HF. Numerous studies have provided proof for the concept that AMPK is protective across diverse cell types in the cardiovascular system.^19^ AMPK can not only improve the energy supply in the failing heart by promoting ATP production but also can regulate several important physiological processes to restore heart function.^20^ Interestingly, MOTS‐c can activate AMPK, and most of the metabolic effects of MOTS‐c treatment are mediated by AMPK activation.^15^ In the present study, we also found that the AMPK pathway was activated in MOTS‐c treated hearts in vivo and in cardiomyocytes transfected with MOTS‐c vector in vitro. Therefore, activation of AMPK in MOTS‐c treated hearts possibly contributed to the improved cardiac function and remodelling. It should be noted that although we found a beneficial effect of MOTS‐c in the heart, the specific effect of MOTS‐c on each type of cell in the heart such as cardiomyocytes, cardiac fibroblasts, endothelial cells, and vascular smooth muscle cells remain unknown, and further studies are needed to address these questions by isolating the primary cell types and testing the effects of MOTS‐c peptide directly in in vitro experiments.

It should be noted that in the present study, the MOTS‐c peptide is derived from humans. Regarding the sensitivity of different species to MOTS‐c treatment, a previous sequence alignment studies of 14 species suggest that MOTS‐c is highly conserved in human, rat, and mouse.^15^ In addition, treatment with human MOTS‐c peptide by intraperitoneal injection in mice has been demonstrated to exert its metabolic‐regulatory effects, such as improved insulin sensitivity, reduced body weight, and prevented high fat diet‐induced obesity and insulin resistance in mice.^15^ Regarding the activity of different form of MOTS‐c, a previous study using HEK293T has showed that cells either stably overexpress MOTS‐c or treated with exogenously with synthetic MOTS‐c could induce global gene expression profile shift and induce the activation of the AMPK pathway compared to the control group.^15^ These results suggest that plasmid transfection with MOTS‐c vector has similar effects as exogenously synthetic MOTS‐c peptide. Consistently, in the present study, we also found that either transfection with plasmid form of MOTS‐c or administration with MOTS‐c peptide could activate the AMPK pathway in the heart both in vitro and in vivo.

Another limitation is that the serum level of MOTS‐c was not determined in the present study. As an endogenous peptide, MOTS‐c has been detected in the serum of mice, rats, and humans. The serum level of MOTS‐c has been reported to be decreased under various stress conditions such as cold exposure, fasted state, and high‐fat‐diet in animal models.^15^, ^21^ In human, circulating level of MOTS‐c was also reported to be reduced in patients with impaired coronary endothelial function and MOTS‐c can improve endothelial function in vitro.^22^ In addition, circulating MOTS‐c levels are also reduced in obese persons and associated with insulin resistance.^23^ In contrast, the serum level of MOTS‐c can be increased by exercise in human.^24^ Taken together, these results suggest that the circulating level of MOTS‐c maybe decreased upon stress or diseased conditions. So, we think that the serum level of MOTS‐c may also be reduced under TAC condition, which needs further verification.

## 5 CONCLUSION

In summary, we provided evidence supporting the therapeutic potential of human MOTS‐c peptides in the prevention and treatment of HF development in the animal model. The beneficial effects of MOTS‐c in HF are possibly attributed to its metabolic‐protective property and, its anti‐inflammatory and anti‐oxidative activities.

## AUTHOR CONTRIBUTIONS

**Peng Zhong:** Conceptualization (equal); writing – review and editing (equal). **Jianye Peng:** Investigation (equal). **Yewen Hu:** Investigation (equal). **Jun Zhang:** Conceptualization (equal). **Caijie Shen:** Investigation (equal).

## CONFLICT OF INTEREST

The authors declare no competing financial interests or conflicts concerning the work described.

## Data Availability

The datasets used and analysed during the current study are available from the corresponding author on reasonable request. All data generated or analysed during this study are included in this published article.
